# Differentiation between primary central nervous system lymphomas and gliomas according to pharmacokinetic parameters derived from dynamic contrast-enhanced magnetic resonance imaging

**DOI:** 10.1016/j.heliyon.2024.e32619

**Published:** 2024-06-06

**Authors:** Yu Zhang, Xiangwei Luo, Youzhi Zhu, Qian Zhang, Bin Liu

**Affiliations:** a Department of Radiology, 901st Hospital of the Chinese People's Liberation Army Joint Logistics Support Force, Hefei, 230031, PR China; b Department of Radiology, The First Affiliated Hospital of Anhui Medical University, Hefei, 230032, PR China

**Keywords:** Primary central nervous system lymphoma, Glioblastoma, Dynamic contrast-enhanced MRI, Pharmacokinetics

## Abstract

**Purpose:**

It is difficult to differentiate between primary central nervous system lymphoma and primary glioblastoma due to their similar MRI findings. This study aimed to assess whether pharmacokinetic parameters derived from dynamic contrast-enhanced MRI could provide valuable insights for differentiation.

**Methods:**

Seventeen cases of primary central nervous system lymphoma and twenty-one cases of glioblastoma as confirmed by pathology, were retrospectively analyzed. Pharmacokinetic parameters, including K^trans^, K_ep_, V_e_, and the initial area under the Gd concentration curve, were measured from the enhancing tumor parenchyma, peritumoral parenchyma, and contralateral normal parenchyma. Statistical comparisons were made using Mann–Whitney *U* tests for V_e_ and Matrix Metallopeptidase-2, while independent samples *t*-tests were used to compare pharmacokinetic parameters in the mentioned regions and pathological indicators of enhancing tumor parenchyma, such as vascular endothelial growth factor and microvessel density. The pharmacokinetic parameters with statistical differences were evaluated using receiver-operating characteristics analysis. Except for the Wilcoxon rank sum test for V_e_, the pharmacokinetic parameters were compared within the enhancing tumor parenchyma, peritumoral parenchyma, and contralateral normal parenchyma of the primary central nervous system lymphomas and glioblastomas using variance analysis and the least-significant difference method.

**Results:**

Statistical differences were observed in K^trans^ and K_ep_ within the enhancing tumor parenchyma and in K_ep_ within the peritumoral parenchyma between these two tumor types. Differences were also found in Matrix Metallopeptidase-2, vascular endothelial growth factor, and microvessel density within the enhancing tumor parenchyma of these tumors. When compared with the contralateral normal parenchyma, pharmacokinetic parameters within the peritumoral parenchyma and enhancing tumor parenchyma exhibited variations in glioblastoma and primary central nervous system lymphoma, respectively. Moreover, the receiver-operating characteristics analysis showed that the diagnostic efficiency of K_ep_ in the peritumoral parenchyma was notably higher.

**Conclusion:**

Pharmacokinetic parameters derived from dynamic contrast-enhanced MRI can differentiate primary central nervous system lymphoma and glioblastoma, especially K_ep_ in the peritumoral parenchyma.

## Highlights

• Pharmacokinetic parameters help to distinguish PCNSL from GBM.
• The Kep in peri-tumoral parenchyma was more effective in diagnosis.
• The signs of PCNSL and GBM were determined by different micro-vascular permeability.
• The microvascular characteristics of PCNSL and GBM were revealed based on pathology.

## 1 Introduction

Compared with glioblastoma (GBM), primary central nervous system lymphoma (PCNSL) is a rare malignant tumor. However, the incidence of PCNSL has increased in recent decades. Both tumors often exhibit solid lump strengthening and invasive growth on conventional magnetic resonance imaging (MRI); however, this poses significant challenges for the differential diagnosis between the two tumors. Furthermore, the treatment and prognosis for PCNSL and GBM differ significantly. Although maximum safe resection is regarded as the standard treatment for GBM, resection in PCNSL is discouraged due to its poor survival benefits and high risk of postoperative deterioration. Stereotactic biopsy followed by chemotherapy is preferred for PCNSL. To provide distinct, specific surgical plans and optimal treatments for GBM and PCNSL, preoperative differential diagnosis is critical [1,2].

In recent years, pharmacokinetic parameters (PPs) derived from quantitative dynamic contrast-enhanced (DCE)-MRI, such as volume transfer constant (K^trans^), rate constant (K_ep_), and volume fraction of extravascular space (V_e_), have emerged as imaging biomarkers of neovasculature and blood–brain barrier (BBB) permeability [3,4]. PPs, especially K^trans^, show a strong correlation with glioma grade [4]. However, there are different opinions on the reliability of using PPs in isolation for PCNSL, and most investigators are interested in enhancing the tumor parenchyma and not the peritumoral parenchyma [[5], [6], [7], [8], [9], [10], [11], [12], [13], [14], [15]](Table 1). Theoretically, in GBM and PCNSL, abnormal peritumoral parenchyma signals are caused not only by changes in interstitial water but also by scattered tumor cell infiltration. Consequently, exploring the peritumoral parenchyma of PCNSL and GBM promises more meaningful information for accurate differential diagnosis and precise delineation of tumor boundaries [16,17]. Hence, in this study, based on DCE-MRI, we attempted to quantitatively analyze the changes in the PPs of the enhancing tumor and peritumoral parenchyma between PCNSL and GBM and explored whether this method could provide useful information for the differential diagnosis of these two tumors.

**Table 1:** References of pharmacokinetic parameter studies comparing PCNSL and GBM.

Study	Sample size	Region of interest	PPs derived from DCE-MRI	Summary of results
Xi YB, Kang XW, Wang N et al., 2019 [5]	PCNSL: 8 HGG: 21	ETP	K^trans^ V_e_	1)K^trans^: PCNSL > HGG, statistical difference 2)V_e_: PCNSL > HGG, statistical difference
Murayama K, Nishiyama Y, Hirose Y et al., 2018 [6]	CNSL: 8 HGG: 15	ETP	K^trans^	30th percentile for K^trans^: CNSL > HGG, statistical difference
Zhang HW, Lyu GW, He WJ et al., 2020 [7]	CNSL: 15 HGG: 28	ETP	K^trans^ K_ep_ V_e_ V_p_ AUC	1) AUC (10th, 25th, median, 75th, 90th, and mean): CNSL > HGG, statistical difference 2) K^trans^, K_ep_, and V_e_ (10th, 25th, median): CNSL > HGG, statistical difference 3) K^trans^, K_ep_, and V_e_ (75th, 90th, and mean): no statistical difference 4) V_p_: no statistical difference
Lu S, Wang S, Gao Q et al., 2017 [8]	PCNSL: 18 GBM: 42	ETP	K^trans^ K_ep_ V_e_ V_p_	1) K^trans^: PCNSL > GBM, statistical difference 2) V_e_: PCNSL > GBM, statistical difference 3) K_ep_ and V_p_: no statistical difference
Lin X, Lee M, Buck O et al., 2017 [9]	PCNSL: 18 GBM: 36	ETP PTP	K^trans^ V_p_	1) 90th percentile of K^trans^ in the ETP: PCNSL vs. GBM, no statistical difference 2) 90th percentile of V_p_ in the ETP: PCNSL > GBM, statistical difference 3) 90th percentile of K^trans^ and V_p_ in the PTP: PCNSL vs. GBM, no statistical difference
Kickingereder P, Sahm F, Wiestler B et al., 2014 [10]	PCNSL: 11 GBM: 60	ETP	K^trans^ K_ep_ V^e^	1) K^trans^: PCNSL > GBM, statistical difference 2) K_ep_: PCNSL > GBM, statistical difference 3) V_e_: no statistical difference
Bhattacharjee R, Gupta M, Singh T et al., 2022 [11]	PCNSL: 48 GBM: 47	ETP	K^trans^ K_ep_ V_e_	1) K^trans^: PCNSL < GBM, statistical difference 2) K_ep_: PCNSL < GBM, statistical difference 3) V_e_: PCNSL > GBM, statistical difference
Saini J, Kumar Gupta P, Awasthi A et al., 2018 [12]	PCNSL: 30 GBM: 70	ETP	K_ep_	K_ep_: PCNSL < GBM, statistical difference
Kang KM, Choi SH, Chul-Kee P et al., 2021 [13]	PCNSL: 25 GBM: 147	ETP	K^trans^ V_e_ V_p_	1) K^trans^: PCNSL vs. GBM, no statistical difference PCNSL > GBM with intermediate or low rCBV, statistical difference 2) V_p_: PCNSL < GBM, statistical difference 3) V_e_: PCNSL > GBM, statistical difference
Lu S, Gao Q, Yu J et al., 2016 [14]	PCNSL: 16 GBM: 38	ETP	K^trans^ K_ep_ V_e_ V_p_	1) K^trans^: PCNSL > GBM, statistical difference 2) V_e_: PCNSL > GBM, statistical difference 3) K_ep_ and V_p_: PCNSL vs. GBM, no statistical difference
Zhao J, Yang ZY, Luo BN et al., 2015 [15]	PCNSL: 6 HGG: 15	ETP PTP	K^trans^ K_ep_ V_e_ iAUC	1) K^trans^ in ETP: PCNSL vs. HGG, no statistical difference 2) V_e_ in ETP: PCNSL > HGG, statistical difference 3) iAUC in ETP: PCNSL > HGG, statistical difference 4) K^trans^ in PTP: PCNSL < HGG, statistical difference 5) V_e_ in PTP: PCNSL > HGG, statistical difference
Our study	PCNSL:17 GBM:21	ETP PTP	K^trans^ K_ep_ V_e_ iAUC	1) K^trans^ and K_ep_ in the ETP: PCNSL < GBM, statistical difference 2) V_e_ and iAUC in the ETP: PCNSL vs. GBM, no statistical difference 3) K_ep_ in PTP: PCNSL < GBM, statistical difference 4) K^trans^, V_e_, and iAUC in the PTP: PCNSL vs. GBM, no statistical difference

PCNSL, primary central nervous system lymphoma; GBM, glioblastoma; HGG, high-grade glioma; ETP, enhancement tumor parenchyma; PTP, peritumoral parenchyma; CNP, contralateral normal parenchyma.

## 2 Materials and methods

### 2.1 Patient information

In total, 38 patients with pathologically confirmed GBM or PCNSL were evaluated retrospectively. All patients with GBM were clinically diagnosed with primary GBM. The exclusion criteria for PCNSL were as follows: a history of extracranial lymphoma; findings of extracranial systemic lymphoma on whole-body imaging screening; identification of mediastinal and/or retroperitoneal lymphadenectasis on imaging studies; findings of lymphoma infiltration with bone marrow biopsy; history of autoimmune disease; positive human immunodeficiency virus status; patients with severe heart, lung, kidney, or liver disease; and a history of present or past illness involving unknown testicular tumors.

All patients underwent routine brain MRI and DCE-MRI before surgical resection or targeted biopsy between 2010 and 2019. This study was approved by the Institutional Ethics Committee of the 901th Hospital of the People's Liberation Army Joint Logistics Support Force (Approval No. 2020090703; approval date:September 10, 2020), and written informed consent was obtained from each patient or the patient's family. This study was conducted in accordance with the principles of the Declaration of Helsinki.

### 2.2 MRI protocol

All patients were scanned using a 3-T scanner (VERIO; SIEMENS Healthcare, Germany) with an eight-channel head coil. The precontrast sequence consisted of an axial T1-weighted image (T1WI), T2-weighted image (T2WI), and fluid-attenuated inversion recovery. All images were acquired with a slice thickness of 5 mm, gap of 1 mm, field of view (FOV) of 24 × 24 cm, and matrix of 256 × 256. The dynamic enhanced scan was acquired using time-resolved imaging with interleaved stochastic trajectories (TWIST). A gadolinium (Gd)-based magnetic resonance (MR) contrast agent (0.2 mL/kg, Magnevist®; Bayer Healthcare Pharmaceuticals, Berlin, Germany) was administered at a rate of 4 mL/s without a preload using an MRI-compatible power injector, followed by a 20 mL saline flush bolus.

The imaging parameters were as follows: repetition time = 3.31 ms, echo time = 1.15 ms, flip angle = 25°, FOV = 30 × 30 cm, matrix = 256 × 230, slice thickness = 5 mm, slab group = 1. A series of 100 dynamic acquisitions were acquired; the first acquisition was 9.6 s, and the remaining acquisitions were 3.8 s each.

T1 mapping is useful for calculating the T1 value of each voxel during the non-contrast phase and has been shown not to alter DCE quantification significantly. However, we did not perform T1 mapping for DCE correction at our institution, as it was not available for image processing in this study. Instead, the contrast medium was injected after the first acquisition [9,18,19]. Contrast-enhanced axial T1WIs were obtained using DCE-MRI.

### 2.3 Imaging post-processing and data analysis

**Fig. 1:**
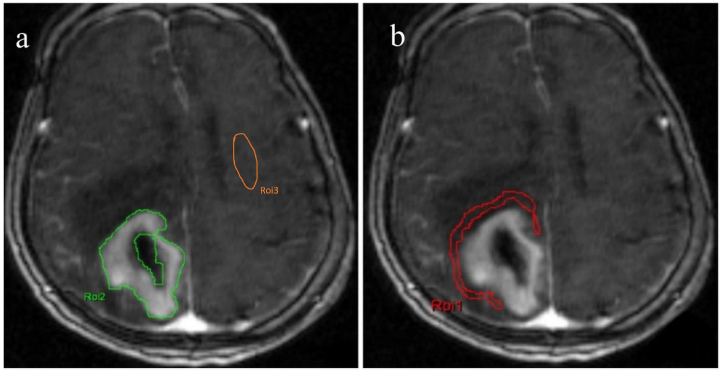
Manual delineation of regions of interest (ROIs). (a) ROIs for the entire enhanced tumor focus at the primary tumor layer and ROIs in the contralateral normal parenchyma, avoiding large blood vessels, cystic changes/necrosis, and the skull. (b) ROIs for peritumoral brain tissues located within 10 mm from the edge of the enhancement foci.

The DCE-T1WI MR images were transmitted for postprocessing using TISSUE 4D software (VB17, SIEMENS Healthineers, Germany), which was included with the scanner. The regions of interest (ROIs) were drawn by two experienced neuroradiologists who were blinded to the diagnosis. In cases of disagreement, a consensus was reached after careful review and modification by a senior neuroradiologist with 20 years of experience. The horizontal segment of the right middle cerebral artery was selected to generate the arterial input function curve. The ROIs were manually drawn on the tumor entity layer in the last stage (the 100th acquisition in DCE-MRI), avoiding large blood vessels, cystic changes/necrosis, and the skull (Fig. 1a). Within the enhancing tumor parenchyma, ROIs were created on a single major enhancement slice on a pixel-by-pixel basis. Within the peritumoral parenchyma, ROIs were circumscribed within 10 mm from the edge of the enhancement foci (Fig. 1b), and within the contralateral normal parenchyma, ROIs were drawn in the center of the half oval (Fig. 1a). Following the methodology outlined in Ref. [20] and the software's operation guidelines, the hemodynamic Tofts two-compartment model was selected. This approach allowed us to generate pseudocolor maps for K^trans^, K_ep_, V_e_, and the initial area value under the Gd concentration curve (iAUC) within the initial 60 s after the injection of the contrast agent. The parameter values were recorded within the enhancing tumor parenchyma, peritumoral parenchyma, and contralateral normal parenchyma of all tumors, and then all data were divided into groups according to the pathology results. The color scale of the pseudocolor maps ranged from blue to red, representing parameter values from low to high.

### 2.4 Histopathology and immunohistochemical analysis

All patients underwent surgical or stereotactic brain biopsy and were diagnosed with PCNSL or GBM. Diagnostic biopsy specimens were obtained from the enhancing tumor parenchyma. The histopathological criteria for GBM encompassed the presence of neoplastic astrocytes with marked nuclear atypia, active mitosis, poor differentiation, and displaying pleomorphic cytomorphology; tumor tissues displaying a high cell density, marked microangiogenesis, and necrosis; and immunohistochemical manifestations characterized by glial fibrillary acidic protein (+) staining.

The histopathological criteria for PCNSL included the absence of neovascularization, minimal interstitial tissue, an abundance of reticular fibers, and occasional necrosis. These tumor cells were uniformly round, tended to cluster around blood vessels, formed a cuff-like arrangement, presented with infiltration along the perivascular space, and tested positive for CD20 (+) and leucocyte common antigen (+) on immunohistochemical analysis.

The expression levels of matrix metalloproteinase-2 (MMP-2), cluster of differentiation 34(CD34), and vascular endothelial growth factor (VEGF) (Santa Cruz, CA, USA) in tumor tissues were semiquantitatively analyzed using Image Pro-Plus 6.0 software (Media Cybernetics, Silver Spring, MD). The mean density (MD) was calculated by measuring the integrated option density and area values of each image. The MD value of three random regions was used as the MD value of this sample. Microvessel density (MVD) was measured based on the expression of CD34. First, the “hot plot” with the highest vascular density was found at low magnification (magnification, × 40–100), and then the number of high-MVD stains in the field of vision was counted at high magnification (magnification, × 400). All pathological evaluations were conducted after consensus by two experienced pathologists.

### 2.5 Statistical analysis

Statistical Package for the Social Sciences 21.0 software was used for statistical analysis. We applied natural logarithm (Ln) transformation to complete the normal distribution conversion. Following a normality test, the quantitative data conforming to a normal distribution were described as mean ± standard deviation, while those that did not conform to a normal distribution were described as the median (25th percentiles, 75th percentiles). In cases with a normal distribution, to compare the K^trans^, K_ep_, V_e_, and iAUC values within the enhancing tumor parenchyma, peritumoral parenchyma, and contralateral normal parenchyma of the PCNSLs and GBMs, analysis of variance and least significant difference tests were performed simultaneously. Additionally, the Bonferroni method was adopted for *P*-value correction. For non-normally distributed data, Wilcoxon rank sum tests were implemented between the paired samples.

The differences between PCNSL and GBM in terms of K^trans^, K_ep_, V_e_, and iAUC values within the enhancing tumor parenchyma, peritumoral parenchyma, and contralateral normal parenchyma regions, tumor cytokine expression levels (VEGF and MMP-2) and tumor MVD values were compared using an independent sample *t*-test. Mann–Whitney *U* tests were implemented when dealing with non-normally distributed data. For parameters displaying statistically significant differences between PCNSL and GBM, we generated receiver operating characteristic (ROC) curves and calculated diagnostic thresholds, sensitivity, and specificity. This study was preliminary and exploratory, and *P*-value correction was performed. A *P*-value <0.05 was considered statistically significant.

## 3 Results

### 3.1 Clinical and pathological findings

GBM was diagnosed in 21 patients (mean age, 56.92 ± 18.67 years; range, 28–83 years; 13 men, 8 women). PCNSL was diagnosed in 17 patients (mean age, 52.86 ± 11.46 years; range, 34–69 years; 12 men and 5 women). According to the aforementioned histopathological criteria, 21 and 17 cases of GBM and PCNSL were classified as World Health Organization grade IV and diffuse large B-cell lymphomas, respectively.

### 3.2 Conventional MRI manifestations of PCNSL and GBM

**Fig. 2:**
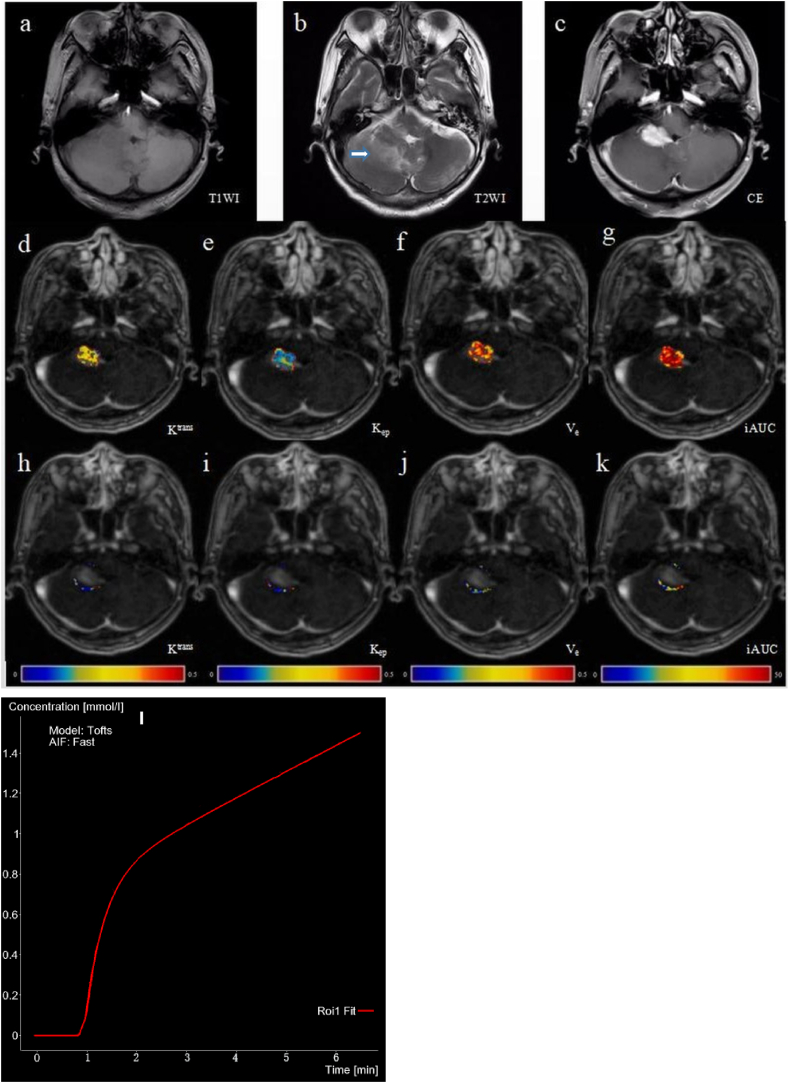
A 59-year-old male with primary central nervous system lymphoma (diffuse large B-cell lymphoma) in the right cerebellum. (a) T1-weighted image showing a slightly low signal. (b) T2-weighted image with a slightly high signal accompanied by peripheral moderate edema. (c) Enhanced scan displaying obvious enhancement. Pseudo-color maps of pharmacokinetic parameters in the enhanced tumor foci: (d) K^trans^ value of 0.232/min and its Ln value was −1.461; (e) K_ep_ value of 0.346/min and its Ln value was −1.061; (f) V_e_ value of 0.690 and its Ln value was −0.371; (g) iAUC value of 47.748 and its Ln value was 3.866. Pseudo-color maps of pharmacokinetic parameters in the peritumoral parenchyma: (h) K^trans^ value of 0.078/min and its Ln value was −2.551; (i) K_ep_ value of 0.185/min and its Ln value was −1.687; (j) V_e_ value of 0.052 and its Ln value was −2.957; (k) iAUC value of 4.964 and its Ln value was 1.602. (l) Fit graph of the enhancing tumor parenchyma based on the fast arterial input function (AIF) of the Tofts model. (For interpretation of the references to color in this figure legend, the reader is referred to the Web version of this article.)

**Fig. 3:**
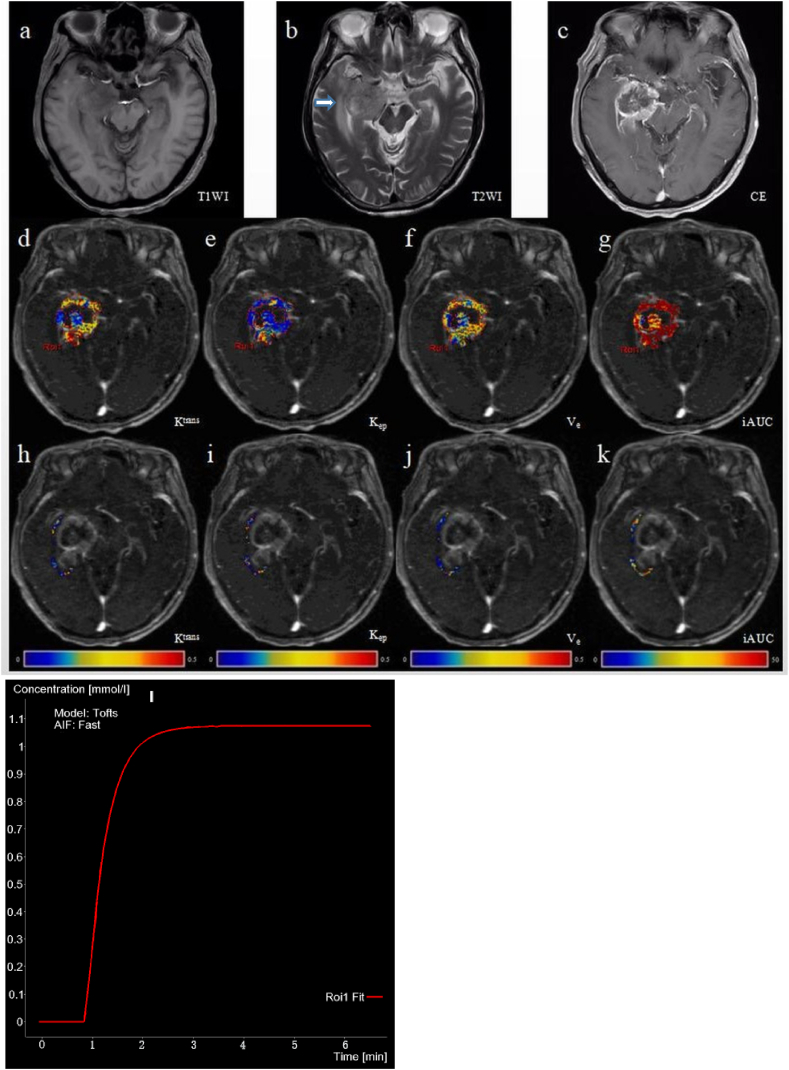
Images of a 74-year-old male with a glioblastoma in the right temporal lobe. (a) T1WI showing slightly low confounding signals. (b) T2WI showing slightly high mixed signals accompanied by peripheral moderate edema. (c) Enhanced scan showing obvious thick ring enhancement. (d ∼ g) Pseudo-color maps of pharmacokinetic parameters in the enhanced tumor foci: K^trans^ value of 0.529/min and its Ln value was −0.637 (d); K_ep_ value of 1.122/min and its Ln value was 0.115 (e); V_e_ value of 0.498 and its Ln value was −0.697 (f); iAUC value of 40.940 and its Ln value was 3.712 (g). (h ∼ k) Pseudo-color maps of pharmacokinetic parameters in the peritumoral parenchyma: K^trans^ value of 0.062/min and its Ln value was −2.781 (h); K_ep_ value of 2.791/min and its Ln value was 1.026 (i); V_e_ value of 0.061 and its Ln value was −2.797 (j); iAUC value of 6.857 and its Ln value was 1.925 (k). (l) Fit graph of the enhancing tumor parenchyma based on the fast arterial input function (AIF) of the Tofts model. (For interpretation of the references to color in this figure legend, the reader is referred to the Web version of this article.)

Mild hypointensity (Fig. 2a) on T1WI and mild hyperintensity (Fig. 2b) on T2WI were observed in 17 cases of PCNSL. Obvious enhancement was evident in 12 cases (Fig. 2c–g), and ring enhancement was evident in five cases. Mild-to-moderate edema was also observed. Slightly low confounding signals (Fig. 3a) on T1WI and slightly high mixed signals (Fig. 3b) on T2WI were observed in 21 cases of GBM. Thick ring enhancement was also evident in 15 cases (Fig. 3c–g), and pronouncedly non-uniform whole-tumor enhancement was present in six cases. Moderate or obvious edema was also observed.

### 3.3 Comparison of PPs between PCNSL and GBM based on DCE-MRI

After taking the Ln, except for the V_e_ in PCNSL in the enhancing tumor parenchyma, which did not conform to a normal distribution, all other parameter values conformed to a normal distribution. As a result, the Ln transformation was discontinued for V_e_ values in PCNSL, and their original data was retained.

In PCNSL, the Ln of K^trans^ in the enhancing tumor parenchyma was slightly higher than that in the peritumoral parenchyma, but no statistical difference was observed (*P* = 0.346, 95 % confidence interval (CI) [−0.206, 0.961]). In addition, the Ln of K^trans^ in the peritumoral parenchyma was higher than that in the contralateral normal parenchyma (*P* < 0.001, 95%CI [1.102, 2.269]) (Fig. 2h–l). The Ln of K_ep_, the Ln of iAUC, and V_e_ value in the enhancing tumor parenchyma were higher than those in the peritumoral (*P* < 0.001, 95%CI [0.638, 1.578]; *P* < 0.001, 95%CI [1.023, 2.145]; Z = −3.432, *P* = 0.001) and contralateral normal parenchymas (Ln of K_ep_: *P* < 0.001, 95%CI [1.207, 2.147]; Ln of iAUC: *P* < 0.001, 95%CI [1.972, 3.094]; V_e_: Z = −3.621, *P* < 0.001). In contrast, the Ln of K_ep_, the Ln of iAUC, V_e_ value in the peritumoral and contralateral normal parenchymas were statistically different (Ln of K_ep_: *P* = 0.013, 95%CI [0.100, 1.039]; Ln of iAUC: *P* < 0.001, 95%CI [0.388, 1.509]; V_e_: Z = −3.575, *P* < 0.001; respectively).

In GBM, the Ln values of K^trans^, V_e_, and iAUC in the enhancing tumor parenchyma were higher than those in the peritumoral (Ln of K^trans^: *P* < 0.001, 95%CI [0.571, 1.392]; Ln of V_e_: *P* < 0.001, 95%CI [1.780, 2.421]; and Ln of iAUC: *P* < 0.001, 95%CI [1.200, 1.918]) and contralateral normal parenchymas (Ln of K^trans^: *P* < 0.001, 95%CI [2.057, 2.878]; Ln of V_e_: *P* < 0.001, 95%CI [2.704, 3.346]; and Ln of iAUC: *P* < 0.001, 95%CI [2.666, 3.250]). Additionally, the Ln values of K_ep_ (*P* < 0.001, 95%CI [1.561, 2.749]) in the peritumoral and contralateral normal parenchymas were significantly different, but no statistical difference was observed (*P* = 0.125, 95%CI [−0.092, 1.096]) between the enhancing tumor parenchyma and peritumoral parenchyma. In contrast, the Ln values of K^trans^, K_ep_, V_e_, and the iAUC in the peritumoral parenchyma were higher than those in the contralateral normal parenchyma (Ln of K^trans^: *P* < 0.001, 95%CI [1.076, 1.896]; Ln of K_ep_: *P* < 0.001, 95%CI [1.561, 2.749]; Ln of V_e_: *P* < 0.001, 95%CI [0.604, 1.245]; and Ln of iAUC: *P* < 0.001, 95%CI [1.040, 1.758]) (Fig. 3h–l) (Table 2).

**Table 2:** Comparison of pharmacokinetic parameters between PCNSL and GBM (mean ± SD or median (25th percentile, 75th percentile).

	Ln of K^trans^ (min^−1^)	Ln of K_ep_ (min^−1^)	Ln of iAUC	Ln of V_e_
				Median
PCNSL (n = 17)				
ETP	−2.26 ± 0.82^a^	−0.92 ± 0.67^a^^,^^b^	2.87 ± 1.00^a^^,^^b^	negligible
				0.617 (0.21, 0.67) a^,^^b^
PTP	−2.64 ± 0.64^a^	−2.03 ± 0.56^a^	1.28 ± 0.35^a^	negligible
				0.046 (0.03, 0.06)
CNP	−4.32 ± 0.58	−2.60 ± 0.39	0.33 ± 0.43	negligible
				0.017 (0.01, 0.02)
GBM (n = 21)				
ETP	−1.73 ± 0.49^a^^,^^b^^,^^c^	0.05 ± 0.87^a^^,^^c^	3.04 ± 0.49^a^^,^^b^	−0.97 ± 0.47^a^^,^^b^
				0.407 (0.29, 0.53)
PTP	−2.71 ± 0.57^a^	−0.45 ± 0.97^a^^,^^c^	1.48 ± 0.55^a^	−3.07 ± 0.42
				0.055 (0.03, 0.07)
CNP	−4.20 ± 0.56	−2.60 ± 0.39	0.09 ± 0.35	−4.00 ± 0.37
				0.017 (0.01, 0.02)

PCNSL, primary central nervous system lymphoma; GBM, glioblastoma; Ln, natural logarithm; ETP, enhancing tumor parenchyma; PTP, peritumoral parenchyma; CNP, contralateral normal parenchyma.

Note:

a Compared within the CNP, *P* < 0.05.

b Compared within the PTP, *P* < 0.05.

c Compared within corresponding regions in PCNSL, *P* < 0.05.

The Ln of K^trans^, the Ln of K_ep_, the Ln of iAUC, and V_e_ values, in the contralateral normal parenchyma were not significantly different between PCNSL and GBM. However, the Ln values of K^trans^ and K_ep_ in the enhancing tumor parenchyma were statistically different between PCNSL and GBM (t = −2.462, *P* = 0.019; t = −3.800, *P* = 0.001); in contrast, the Ln values of iAUC, V_e_ values did not differ significantly (t = −0.665, *P* = 0.513; Z = −1.541, *P* = 0.128). Meanwhile, the Ln of K_ep_ in the peritumoral parenchyma was statistically different between PCNSL and GBM (t = −6.298, *P* < 0.001), whereas the Ln of K^trans^, V_e_ values, and the Ln of iAUC were not statistically different (Ln of K^trans^: t = 0.397, *P* = 0.694; V_e_: Z = −0.632; *P* = 0.542, Ln of iAUC: t = −1.372, *P* = 0.179).

### 3.4 ROC curve analysis of PPs between PCNSL and GBM

**Fig. 4:**
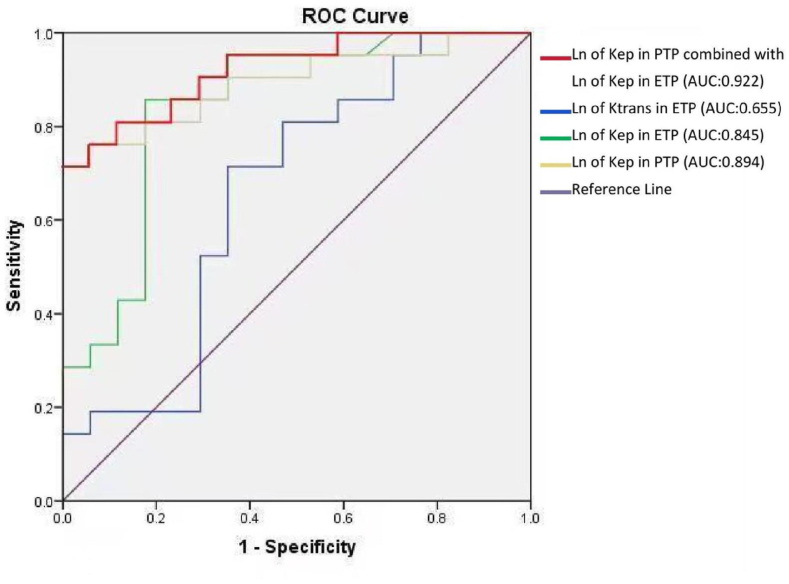
ROC curve for pharmacokinetic parameters between primary central nervous system lymphoma and glioblastoma. ETP, enhancing tumor parenchyma; PTP, peritumoral parenchyma.

Following the identification of statistically significant differences, ROC curve analysis was used to further compare the diagnostic performance of log-transformed K^trans^ and log-transformed K_ep_ in the enhancing tumor parenchyma and K_ep_ in the peritumoral parenchyma between PCNSL and GBM (Table 3). Compared with log-transformed K^trans^ in the enhancing tumor parenchyma, the Ln of K_ep_ in the enhancing tumor parenchyma and Ln of K_ep_ in the peritumoral parenchyma had higher diagnostic efficiency, specificity, and sensitivity. Notably, the Ln of K_ep_ in the enhancing tumor parenchyma and the Ln of K_ep_ in the peritumoral parenchyma had good diagnostic values, and the AUCs of both were above 0.8 (Fig. 4).

**Table 3:** ROC curve analysis of pharmacokinetic parameters between PCNSL and GBM.

Parameters	Area under the curve	*P*-values	Diagnostic threshold	Specificity	Sensitivity
Ln of K^trans^ in ETP	0.655	0.103	−1.928	64.71 %	71.43 %
Ln of K_ep_ in ETP	0.845	<0.001	−0.716	82.36 %	85.71 %
Ln of K_ep_ in PTP	0.894	<0.001	−0.821	100.00 %	71.43 %
Ln of K_ep_ in PTP combined with Ln of K_ep_ in ETP	0.922	<0.001	–	100.00 %	71.43 %

ROC, receiver operating characteristic; PCNSL, primary central nervous system lymphoma; GBM, glioblastoma; ETP, enhancing tumor parenchyma; PTP, peritumoral parenchyma.

**Table 4:** Comparison of cytokine expression levels and MVD between PCNSL and GBM.

	MMP2 (IOD/area) median (25th percentile, 75th percentile）	VEGF (IOD/area) mean ± SD	MVD mean ± SD
PCNSL (n = 17)	0.0422 (0.0262, 0.0700)	0.0347 ± 0.022	9.29 ± 4.74
GBM (n = 21)	0.0865 (0.0669, 0.1013)	0.0874 ± 0.038	30.33 ± 15.21
	F = −2.951	t = −5.103	t = −5.477
	*P* = 0.003	*P* < 0.001	*P* < 0.001

PCNSL, primary central nervous system lymphoma; GBM, glioblastoma; VEGF, vascular endothelial growth factor; MMP2, matrix metalloproteinase-2; MVD, microvessel density; IOD, integrated option density. *P* < 0.05 was considered statistically significant.

The diagnostic threshold was calculated according to the ROC curve analysis. When the Ln of K_ep_ in the enhancing tumor parenchyma was >-0.716, the specificity of the diagnosis of GBM was 82.36 %, with a sensitivity of 85.71 % and AUC was 0.845. Conversely, when the Ln of K_ep_ in the peritumoral parenchyma was >-0.821, the specificity was 100.00 % and the sensitivity was 71.43 % and AUC was 0.894. When combining the Ln of the K_ep_ of the peritumoral parenchyma and the Ln of the K_ep_ of the enhancing tumor parenchyma, the specificity was 100.00 %, with a sensitivity of 71.43 % and AUC was 0.922.

### 3.5 Comparison of cytokine expression levels and MVD between PCNSL and GBM (Table 4)

**Fig. 5:**
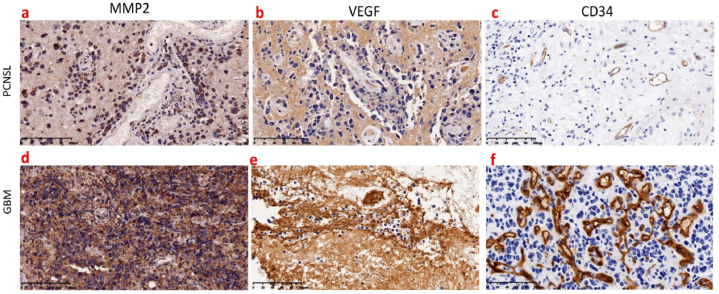
Histopathology of primary central nervous system lymphoma (PCNSL) and glioblastoma (GBM) in the same patients as shown in Fig. 2, Fig. 3. (a) In PCNSL, the mean density (MD) of matrix metalloproteinase-2 (MMP-2) expression was 0.0210; (b) the mean density (MD) of vascular endothelial growth factor (VEGF) expression was 0.0361; and (c) the microvessel density (MVD) of cluster of differentiation 34 (CD34) expression was 10. (d) In glioblastoma (GBM), the mean density (MD) of matrix metalloproteinase-2 (MMP-2) expression was 0.1551; (e) the mean density (MD) of vascular endothelial growth factor (VEGF) expression was 0.1533; and (f) the microvessel density (MVD) based on cluster of differentiation 34 (CD34) expression was 39.

Except for the MMP-2 expression levels in PCNSL, which did not conform to a normal distribution, all other cytokine expression levels conformed to a normal distribution. In the enhancing tumor parenchyma, the expression levels of MMP-2 (Fig. 5a, F = −2.951, *P* = 0.003) and VEGF in PCNSL (Fig. 5b, t = −5.103, *P* < 0.001), as well as MVD (Fig. 5c, t = −5.477, *P* < 0.001), were lower than those in GBM (Fig. 5d ∼ f), and these differences were statistically significant.

## 4 Discussion

DCE-MRI is a dynamic scan technique that relies on a rapid imaging sequence to obtain physiological information related to the distribution of a contrast agent in the capillary network and interstitial spaces, which can reflect changes in tumor microcirculation perfusion and capillary permeability [21]. In our study, we employed the TWIST protocol, which utilizes a three-dimensional fast gradient-recalled echo to capture images from the k-space center (A) region and differently undersampled k-space periphery (B) regions. This DCE-MRI method not only significantly improves temporal resolution but also preserves excellent spatial resolution. Therefore, this fast and high spatial resolution DCE imaging method was well-suited for pharmacokinetic analysis [22].

Through mathematical operations, various PPs were acquired, including K^trans^, K_ep_, and V_e_ [9]. While these parameters are primarily indicative of vascular permeability, among which the K^trans^ value is considered to be the most stable parameter, these were also affected by various vascular physiological factors, including blood flow velocity, vascular permeability, tumor vessel density, vascular bed area, and extravascular extracellular space (EES). Owing to the integrity of the BBB, the K^trans^ in the contralateral normal parenchyma tends to be close to [21,23,24], and our data also demonstrated a similar result on the normal side.

Our data showed that the K^trans^ and K_ep_ values of PCNSL and GBM were statistically different. In addition, in our study, the MVD of PCNSL was lower than that of GBM in the enhancing tumor parenchyma, and there was a statistical difference between the two. Similarly, Liao et al. found that the CD43 levels and MVD of PCNSL were lower than those of high-grade gliomas (HGGs) [25]. Other researchers have also considered that PCNSLs are hypovascular and markedly strengthened because of their high permeability [26]. In contrast, GBMs are highly vascularized malignant tumors, and their enhancement is a result of both tumor angiogenesis and disruption of the BBB [27]. Additionally, our data showed that the levels of VEGF and MMP-2 in GBM were higher than those in PCNSL, suggesting that VEGF could affect the permeability of the BBB [28]. It is important to note that MMP-2 is a member of the MMP family, which are a group of key enzymes involved in the degradation of the extracellular matrix during cellular invasion and permeability control of the BBB, and may be associated with the prognosis of PCNSL [29,30]. While this data alone may not definitively confirm that GBM has consistently higher permeability than PCNSL, it does indicate the possibility and strengthens the association. Furthermore, we hypothesized that the BBB disruption, combined with the presence of numerous immature blood vessels and elevated VEGF and MMP-2 expression in GBM tissues, resulted in a higher MVD, larger vascular bed area, faster blood flow velocity, and increased tumor vascular permeability, which may have caused leakage of the contrast agent molecules from the blood vessels. The heightened permeability, influenced by multiple factors, likely determined both the leakage velocity and quantity of contrast agent molecules leaked, with the exchange rate into the blood vessel surpassing that of PCNSL. In addition, the abundant network fibers in the PCNSL tissue led to the slower penetration and backflow of the contrast agent [31], explaining the differences in K^trans^ and K_ep_ values between PCNSL and GBM, with GBM parameters being higher.

Existing studies have reported inconsistent findings regarding the differences in PPs derived from DCE-MRI between these two tumor types. Similar to our results, some studies have demonstrated that the microvascular permeability constants of PCNSL, such as the K^trans^, and/or K_ep_ values, were lower than those of GBM or HGG [11,12,15]. In contrast, some studies have demonstrated that there is no difference in the K^trans^ or K_ep_ of DCE-MRI between the two tumors [[7], [8], [9],^13^,^14^]. In contrast, others demonstrated that in PCNSL or CNSL, the K^trans^ and/or K_ep_ values were significantly higher than those in GBM or HGG [[5], [6], [7], [8],^10^,^13^,^14^]. This phenomenon may be related to differences in the algorithms and sequences employed by different centers, the intrinsic heterogeneity of tumors, the elusive complexity of the microvascular hemodynamics in GBM, and variability in the status of the disrupted BBB in PCNSL. Meanwhile, Kickingereder et al. [10] conducted a comparative analysis of the microvascular morphology between PCNSL and GBM using histopathological methods, attempting to elucidate the reasons behind the differences in parameter values between the two tumor types. Regrettably, akin to our study, they did not employ an appropriate histopathological quantitative analysis technique to determine the extent of BBB disruption but rather provided descriptive and theoretical explanations for these observations [10].

The existing literature indicates that studies focusing on the peritumoral parenchyma based on the PPs of DCE-MRI are limited. In previous studies, researchers suggested that these two types of tumors could infiltrate the peritumoral brain tissue, with neovascularization being present in the peritumoral tissues of GBMs [16,17,32,33], while the infiltration of PCNSL into the peritumoral tissue mainly results in the destruction of the integrity of the BBB [27]. Unfortunately, we did not conduct a histopathological control study of the peritumoral brain tissue due to a lack of preoperative planning and ethical considerations. In our study, there were statistically significant differences in the K^trans^ values of PCNSL and GBM between the peritumoral brain and contralateral normal parenchyma, indicating that the microvascular characteristics of the peritumoral brain tissues had changed. Notably, this observation aligns with the findings of previous pathophysiological imaging studies. Interestingly, unlike the enhanced tumor foci, our data showed no difference in K^trans^ and revealed a significant difference in K_ep_ values between the peritumoral parenchyma of PCNSL and that of GBM, which may indicate that although both PCNSL and GBM invaded the surrounding brain tissue, the extent and manner of destruction may not be exactly the same [28,34,35]. Ultimately, the changes in microvascular characteristics caused by PCNSL and GBM infiltrating the peritumoral parenchyma were minimal [28,[32], [33], [34], [35]]. Consequently, the parameter values for the peritumoral parenchyma in our data closely resembled those of the contralateral normal parenchyma.

Based on the minimal infiltration observed, we speculated that there might be no difference in the state of the molecules flowing out of the microvasculature in the peritumoral parenchyma of the two tumors. However, angiogenesis is generated by GBM infiltrating the peritumoral parenchyma, which promotes the reflux of contrast agent molecules. This may explain why the K_ep_ value of the peritumoral parenchyma was higher in GBM than in PCNSL. Similarly, Zhao et al. found that both the K_ep_ and K^trans^ of HGG were higher than those of PCNSL in the peritumoral parenchyma [15]. In contrast, Lin et al. found no statistically significant differences in the PPs of the peritumoral parenchyma [9]. Therefore, there is a contradiction in the quantitative parameter analysis of vascular permeability between PCNSL and GBM based on imaging methods in clinical research. Indeed, it may be necessary to gather evidence by conducting animal experiments on PCNSL to establish the correlation between the histopathology of BBB permeability and the molecular proteins related to permeability, which could help validate the aforementioned hypothesis.

V_e_ is equal to the ratio of K^trans^ over K_ep_ (V_e_ = K^trans^/K_ep_), and it is not an independent parameter. In our data, we found no statistical difference in V_e_, which represents the space capacity of the EES, between the enhancing tumor and peritumoral parenchyma of both PCNSL and GBM. This suggests that both PCNSL and GBM, being highly malignant tumors with high cell density, may have a similar EES space capacity. This finding aligns with the results reported by Kickingereder et al. [10]. However, the values of V_e_ were inconsistent in similar studies. For example, Zhao et al. found that the V_e_ values of PCNSL were higher than those of HGG in both the enhancing tumor and peritumoral parenchyma [15]. They concluded that V_e_ was positively correlated with the volume of the trapped contrast agent in the tumor interstitium. In other words, the greater the confinement of the contrast agent in the tumor interstitium, the higher the V_e_ value. In PCNSL, the contrast agent is constrained by a less pronounced disruption of the BBB and/or perivascular lymphocytic cuffs [15]. Interestingly, this theoretical explanation by Zhao et al. might also shed light on the observation that K_ep_ was lower in PCNSL than in GBM in our study.

The iAUC is a semiquantitative analysis parameter that is derived from the signal-time curve, which is the sum of the area under the time-signal curve during the initial scanning time, and this parameter is related to various pathophysiological factors within the tumor. It mainly reflects the blood volume of tumor foci during a particular period of dynamic enhancement [36]. We found that compared to the contralateral normal parenchyma, the iAUC values in the peritumoral parenchyma of GBM and PCNSL differed significantly, possibly indicating that the blood volume in the peritumoral parenchyma of both tumor types increased and indirectly confirming the presence of microvascular changes in the peritumoral parenchyma.

In our data, there were no differences in iAUC between GBM and PCNSL in the enhancing tumor parenchyma, possibly due to the substantial enhancement observed in both tumors. However, previous studies by Zhao and Zhang et al. reported significantly higher iAUC values in PCNSL and CNSL [7,15]. They proposed that the histological features of PCNSL, involving infiltrated lymphatic cells forming networks around arterioles and venules, contributed to severe enhancements. Conversely, in Choi et al.’s study employing DCE-MRI, the iAUC values were lower in PCNSL than in GBM [37]. Interestingly, based on the ROC curve analysis, we found that among the parameters with statistical differences between GBM and PCNSL, the parameters with high diagnostic efficacy were not in the enhancing tumor parenchyma but rather K_ep_ in the peritumoral parenchyma. While Zhao et al. found similar results, they believed that K^trans^ in the peritumoral parenchyma was more effective for diagnosis [15].

In practice, the pixel-to-pixel analysis method is difficult to implement because it requires an exact slice match. Even a small shift can be problematic, especially in ROIs with large heterogeneity [20]. In such cases, histograms of PPs may prove valuable in analyzing the distribution of the Gd uptake rates in the tumor region [20]. Nonetheless, this preliminary study demonstrated an advantage in using this approach. Therefore, it is advisable to conduct multicenter studies with larger sample sizes and explore histogram-based methods further to enhance research techniques.

## 5 Conclusions

While we acknowledge that our study's small sample size might introduce some bias, these results serve as a preliminary indication that there are notable distinctions in the PPs derived from DCE-MRI between PCNSL and GBM. Specifically, alterations in certain PPs were observed in the peritumoral parenchyma of both tumors. The differences between PCNSL and GBM were determined based on their different microvascular permeabilities and the distribution of contrast agents within and outside the blood vessels. The findings suggest that PPs derived from DCE-MRI can be useful for distinguishing between PCNSL and GBM, potentially aiding in the differential diagnosis of these brain tumors.

## Data availability statement

All data used in the generation of the results presented in this manuscript will be made available upon reasonable request from the corresponding author.

## Funding

The 901st Hospital Project Fund of the Chinese People's Liberation Army Joint Logistic Support Force (No. 2023YGZD08).

10.13039/501100017668Key Research and Development Program of Anhui Province (No. 1804h08020284).

## CRediT authorship contribution statement

**Yu Zhang:** Writing – review & editing, Writing – original draft, Methodology, Formal analysis, Conceptualization. **Xiangwei Luo:** Writing – review & editing, Writing – original draft, Methodology, Funding acquisition, Data curation. **Youzhi Zhu:** Funding acquisition, Formal analysis, Conceptualization. **Qian Zhang:** Methodology, Formal analysis, Data curation. **Bin Liu:** Writing – review & editing, Project administration, Methodology, Formal analysis.

## Declaration of competing interest

The authors declare that they have no known competing financial interests or personal relationships that could have appeared to influence the work reported in this paper.
